# A Neonatal Case of Glial Choristoma of the Tongue Causing Airway Obstruction

**DOI:** 10.1155/2017/2413035

**Published:** 2017-09-20

**Authors:** Hajime Machi, Hiroki Karata, Yusuke Yamane, Junya Fukuoka, Yasutomo Funakoshi, Hiroyuki Moriuchi

**Affiliations:** ^1^Department of Pediatrics, Nagasaki University Hospital, 1-7-1 Sakamoto, Nagasaki, Japan; ^2^Department of Pathology, Nagasaki University Hospital, 1-7-1 Sakamoto, Nagasaki, Japan; ^3^Department of Pediatric Surgery, Nagasaki University Hospital, 1-7-1 Sakamoto, Nagasaki, Japan

## Abstract

Glial choristoma is considered to be a type of brain heterotopia consisting of ectopic central nervous tissue. We herein report a neonate with glial choristoma of the tongue who developed respiratory distress due to airway obstruction. A male neonate presented with respiratory distress due to a soft mass on the midline region of the dorsal tongue base at birth. He was intubated using a flexible fiberoptic nasopharyngoscope. MRI showed a well-circumscribed mass measuring 25 × 23 × 27 mm in size in the same region. A histologic examination confirmed a pathological diagnosis of glial choristoma. He underwent tracheotomy at 22 days of age, and a subtotal resection of the tumor was performed at five months of age. The clinical behavior of oral glial choristoma varies depending on the age at onset as well as the location and size of the mass. The small size of the organ and the narrow operating field hamper the surgical approach in neonates. The optimal therapeutic strategy for neonatal cases of glial choristoma should thus be determined based on the condition of each individual patient.

## 1. Introduction

Choristoma generally consists of heterotopic tissue that is microscopically normal. Glial choristoma is considered to be a type of brain heterotopia that comprises ectopic central nervous tissue. It usually presents in the head and neck region. The nasal area is most often affected [1], and the occurrence of glial choristoma of the oral region, most frequently occurring in the palatopharyngeal complex area, is relatively rare [2]. Glial choristoma presenting as a tongue lesion, which occurs less frequently than that in the palatopharyngeal complex area, is also rarely associated with minor breathing and feeding problems [3].

We herein report a neonate who presented with glial choristoma of the tongue who developed respiratory distress due to airway obstruction.

## 2. Case Presentation

A male neonate was born by normal vaginal delivery at 37 weeks of gestation. His birth weight was 3010 g (72th percentile), height 48 cm (58th percentile), and head circumference 35 cm (95th percentile). His 29-year-old mother had gestational diabetes mellitus controlled by the dietary treatment. Polyhydramnios had been detected by ultrasonography at 33 weeks of gestation. Neither fetal ultrasonography nor magnetic resonance imaging (MRI) revealed any abnormal findings accounting for polyhydramnios.

**Figure 1:**
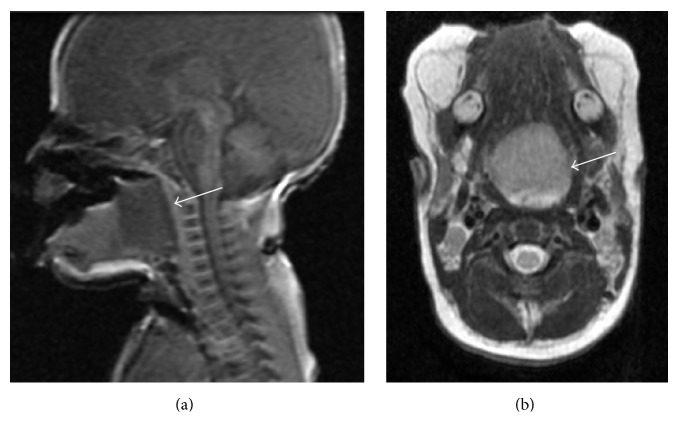
MRI findings of the mass on the tongue base. MRI shows a well-circumscribed mass measuring 25 × 23 × 27 mm in size in the midline region of the tongue base with a low signal intensity on T1-weighted images (a) and a high signal intensity on T2-weighted images (b).

He presented with symptoms of respiratory distress associated with tachypnea, cyanosis, and retractive breathing at birth. Even when the baby attempted to cry, his voice could barely be heard. An immediate observation of his oral cavity identified a soft mass on the midline region of the dorsal tongue base. Due to progressive respiratory distress with marked hypercapnia, he was intubated using a flexible fiberoptic nasopharyngoscope, because the mass was prone to bleeding by contact. Contrast-enhanced computed tomography (CT) imaging revealed a low-density, nonenhancing 20 mm wide mass on the midline region of the tongue base. MRI showed a well-circumscribed mass measuring 25 × 23 × 27 mm in size in the same region with a low signal intensity on T1-weighted images (Figure 1(a)) and a high signal intensity on T2-weighted images (Figure 1(b)).

**Figure 2:**
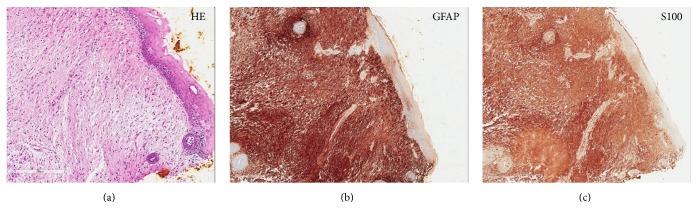
The histopathological findings of the mass. Hematoxylin and eosin (H&E) staining of the tissue specimen shows a subepithelial eosinophilic fibrous mass without a capsule (a). Immunostaining for GFAP (b) and S100 (c) was positive.

A biopsy was performed at 15 days of age. A histological analysis of the biopsy specimens revealed a subepithelial eosinophilic fibrous mass without a capsule. Mature glial tissue characterized by spindle-shaped cells with eosinophilic cytoplasm and slight myxoid change without atypia were observed (Figure 2(a)). The cellularity was homogenously low, and no mitosis or necrosis was identified. Immunohistochemical staining for glial fibrillary acid protein (GFAP) (Figure 2(b)) and S100 (Figure 2(c)) were diffusely positive for a spindle cell component. Based on these findings, a pathological diagnosis of glial choristoma was made.

Curative surgery during the neonatal period was deemed too risky, so he underwent tracheotomy at 22 days of age. Since his difficulty in swallowing had probably been caused by the tongue mass, he was fed through a nasogastric tube. His respiratory condition remained stable except for short-term hospitalization due to acute bronchitis at four months of age, when the mass on MRI was noted to be almost the same size as that observed at birth.

He underwent subtotal resection of the mass at five months of age. The postoperative movement of the tongue and the vocal cords during phonation were good. Although he was able to swallow milk and solid foods, he remained dependent on nasal feeding due to the slight aspiration of liquid, probably resulting from a very mild complication affecting the movement of the epiglottis.

## 3. Discussion

Glial choristomas are rare congenital head and neck lesions seen most commonly in neonates or in early childhood. They consist of differentiated neuroectodermal tissue and represent developmental heterotopias rather than true neoplasms [4]. The nasal area is most often affected with approximately 250 cases reported [1, 5], and glial choristoma of the oral region is relatively rare with only approximately 30 cases reported [2, 5]. A review of the literature on oral glial choristoma showed the most frequently reported site to be the palatopharyngeal complex area (17 [55%] of 31 cases), followed by the tongue (11 [35%]) [3]. The clinical behavior of oral glial choristoma varies by the age at onset as well as the location and size of the tumor. While most such tongue lesions do not cause any complications, palatopharyngeal masses are frequently symptomatic and sometimes life-threatening because of potential airway obstruction and serious feeding problems, especially in neonates. Tracheostomy and endotracheal intubation were performed in some of those cases to enable the patients to overcome acute respiratory distress [3, 6, 7].

Although a surgical resection is the standard treatment for glial choristoma, the optimal timing for surgery remains unclear and probably depends on the condition of each patient. While early resection enables extubation and oral feeding [8], the small size of the organ and the narrow operating field hamper the surgical approach. Glial choristomas are generally present at birth or manifest within the first few years of life, and most grow slowly at a rate parallel to the surrounding tissue in an expansive fashion [2]. In the present case, respiratory and nutritional management with tracheotomy and nasal feeding stabilized the patient's condition, and the glial choristoma did not increase in size for approximately four months; therefore, an invasive operation in this case was able to be safely postponed until five months of age.

Glial choristoma should be considered in the differential diagnosis of neonatal airway obstruction. It may be best to determine the therapeutic strategy for neonatal cases of glial choristoma based on the condition of each individual patient.
